# Evidence for Isolation-by-Habitat among Populations of an Epiphytic Orchid Species on a Small Oceanic Island

**DOI:** 10.1371/journal.pone.0087469

**Published:** 2014-02-03

**Authors:** Bertrand Mallet, Florent Martos, Laury Blambert, Thierry Pailler, Laurence Humeau

**Affiliations:** 1 UMR Peuplements Végétaux et Bio-Agresseurs en Milieu Tropical, Université de La Réunion, Saint-Denis, Ile de La Réunion, France; 2 School of Life Sciences, University of KwaZulu-Natal, Pietermaritzburg, South Africa; Tuscia University, Italy

## Abstract

Identifying factors that promote population differentiation is of interest for understanding the early stages of speciation. Gene flow among populations inhabiting different environments can be reduced by geographical distance (isolation-by-distance) or by divergent selection resulting from local adaptation (isolation-by-ecology). Few studies have investigated the influence of these factors in small oceanic islands where the influence of geographic distance is expected to be null but where habitat diversity could have a strong effect on population differentiation. In this study, we tested for the spatial divergence of phenotypes (floral morphology and floral scent) and genotypes (microsatellites) among ten populations of *Jumellea rossii*, an epiphytic orchid endemic to Réunion growing in three different habitats. We found a significant genetic differentiation between populations that is structured by habitat heterogeneity rather than by geographic distance between populations. These results suggest that ecological factors might reduce gene flow among populations located in different habitats. This pattern of isolation-by-habitat may be the result of both isolation-by-ecology by habitat filtering and asynchrony in flowering phenology. Furthermore, data on floral morphology match these findings, with multivariate analysis grouping populations by habitat type but could be only due to phenotypic plasticity. Indeed floral scent compounds were not significantly different between populations indicating that specific plant-pollinator mutualism does not seem to play a major role in the population differentiation of *J. rossii.* In conclusion, the results from our study emphasize the importance of habitat diversity of small oceanic islands as a factor of population differentiation.

## Introduction

Genetic differentiation among populations of a species can be the result of limited gene flow and genetic drift followed by divergent natural selection acting on these different gene pools [1]–[3]. On the contrary, a strong disruptive selection can initiate divergence among populations through local adaptation, which finally reduce gene flow by selection against migrants or assortative mating [4], [5]. In this situation, drift can increase population divergence by causing linkage disequilibrium between selected and reproductive traits [6]. Population differentiation is therefore an evolutionary split combining neutral and non-neutral processes; however, their relative significance is not in general agreement in the literature [7], [8].

So far, population differentiation has been mainly studied in a geographical context in which initial divergence between populations depends on the level of migration between them [2], [3], [9]. Dispersal probability is expected to decrease with distance so that gene flow is generally lower between geographically distant populations causing a pattern of isolation-by-distance (IBD, [2]). Besides IBD, geographically close populations may also diverge because of the great ecological contrast among them; in this case, the level of gene flow would decrease between populations in different environments causing a pattern of isolation-by-ecology (IBE, we always use this generic term as defined in [4]). A correlation between genetic divergence and geographical distance is expected under IBD, whereas a correlation between genetic divergence and environmental dissimilarity is expected under IBE. However, geographical and environmental factors are not exclusive and can act together to reduce gene flow between populations [10]–[12].

Species exclusively found on small oceanic islands are excellent systems to study ecological isolation because geographical distances between populations are a priori less important on a small oceanic island than on the mainland. Moreover young oceanic islands are topographically complex and, as a result, a wide range of climatic and ecological conditions is found at small geographic scale [13]. For instance, Milá et al. [14] showed a strong morphological and genetic differentiation among populations of a passerine bird in the Mascarene archipelago, which occurred along an altitudinal gradient of habitat types principally, despite short geographic distances separating them. This case study suggests that isolation-by-ecology may be an important driver for intra-island population differentiation, although this needs be further investigated in other organisms.

Orchids are particularly interesting in this context because they produce numerous minute seeds capable of long-range dispersal [15] and are thus generally well represented on remote oceanic islands. Even though intra-island migrations might occur continuously, suitable mycorrhizal fungi are nevertheless required for orchid germination and seedling establishment, as well as suitable pollinators population maintenance [16], [17]. Moreover, orchids usually have small effective population sizes, which may stress the effect of genetic drift [18], [19]. However, Phillips, Dixon and Peakall [20] showed that this family tends to exhibit low levels of population differentiation, especially at small spatial scale, and suggested that drift might not play a major role in orchid population differentiation and speciation. Nevertheless, they pointed out that very few studies have been conducted in tropical epiphytic orchids and consequently emphasized the need for future research on these groups.

Here, we focus on the genetic differentiation among populations of an epiphytic orchid species, *Jumellea rossii*, which is endemic to Réunion and widespread across several habitat types on this island [21]. Moreover, given that this species shows some obvious phenotypic variation across its range [22], we also investigate the population differentiation for some phenotypic traits (i.e. floral morphology and scent chemistry). The aim of this study is to assess the relative significance of geographic and environmental distances in isolating populations of *J. rossii* in Réunion.

## Materials and Methods

### Study area and species

**Figure 1:**
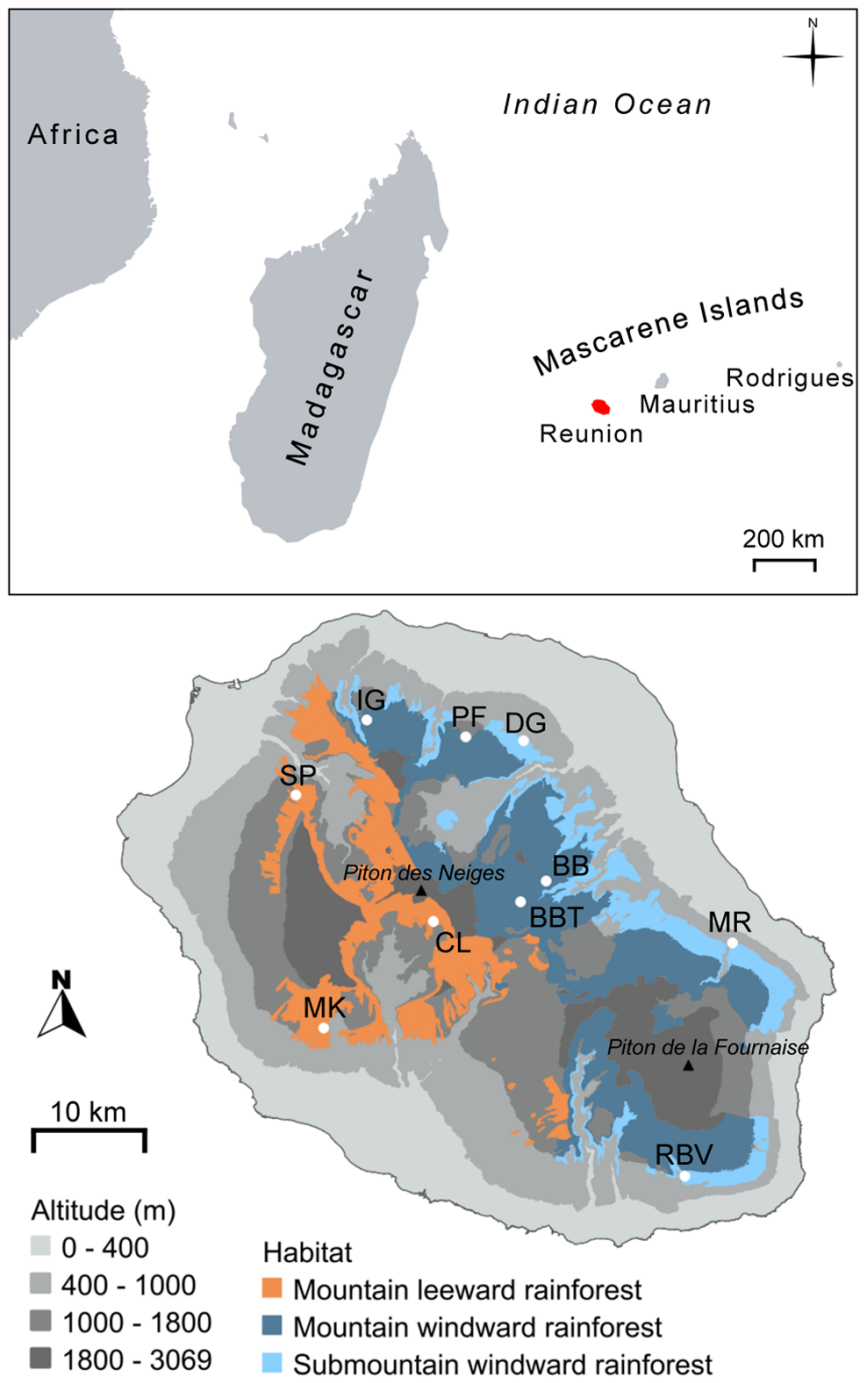
Location of Réunion and study populations. The top map shows the location of Réunion (in red) in the southwest Indian Ocean. The bottom map shows the location of study populations and the distribution of the three natural habitats types of *Jumellea rossii*.

Réunion (55°39′E; 21°00′S) is a small (2512 km^2^) and young (about two million years) oceanic island in the Mascarene archipelago. Two volcanic massifs shape the island; one of them is still active (Piton de la Fournaise; Fig. 1), which confers to Réunion a complex topography and a resulting strong variation in rainfall from east (wet) to west (dry) and in temperature along the altitudinal gradient. As a result, Réunion is ecologically heterogeneous and has got about 20 well-distinct habitat types despite its small surface area [23]. Orchids represent approximately 20% of the native vascular flora, and species composition, breeding systems and floral traits vary along the altitudinal gradient principally [24].

*Jumellea rossii* Senghas is a long-lived perennial orchid that is exclusively found in the wetter forests of Réunion between 500 and 1800 m a.s.l. Populations grow epiphytically in three main habitat types: mountain windward rainforest (MWR), mountain leeward rainforest (MLR), and submountain windward rainforest (SWR; Table 1, Fig. 1). Autonomous self-pollination is unlikely, but *J. rossii* is self-compatible and geitonogamous pollination seems to be frequent in this orchid that rewards moth pollinators with a copious amount of nectar contained in a long spur (Mallet, unpublished data). Pollinators include at least one Sphingidae and two Noctuidae species (Mallet, unpublished data). Plants form dense clumps of 20–50 cm high stems, each stem producing one to five flowers, white in colour, and each flower emitting a pleasant, sweet fragrance at dusk. The flowering period is between December and March during the rainy season, and variation in floral phenology remarkably correlates with altitude: highland populations flower earlier than lowland populations. A molecular phylogeny of the genus *Jumellea* [25] showed that within-island speciation events occurred in Réunion for several lineages including *J. rossii*.

**Table 1:** Characteristics of the studied populations of Jumellea rossii in Réunion.

Population	Code	Longitude	Latitude	Altitude	*N*_m_	*N*_g_	*N*_c_
**Mountain Windward Rainforest**	**MWR**				**40**	**173**	**3**
Plaine des Fougères	PF	55°31′04″E	20°58′29″S	1220 m	14	23	–
Bébour	BB	55°35′23″E	21°05′52″S	1140 m	11	50	–
Bébour-Takamaka	BBT	55°33′53″E	21°06′32″S	1370 m	15	48	3
Ilet à Guillaume	IG	55°25′01″E	20°57′38″S	1200 m	–	52	–
**Mountain Leeward Rainforest**	**MLR**				**42**	**124**	**5**
Cilaos	CL	55°29′21″E	21°07′27″S	1400 m	25	40	3
Saint-Paul	SP	55°22′26″E	21°01′14″S	1390 m	17	50	2
Les Makes	MK	55°23′36″E	21°12′35″S	1120 m	–	34	–
**Submountain Windward Rainforest**	**SWR**				**51**	**113**	**8**
Mourouvain	MR	55°44′52″E	21°08′37″S	550 m	16	21	2
Dugain	DG	55°34′07″E	20°58′42″S	770 m	20	52	3
Basse-Vallée	RBV	55°42′16″E	21°19′30″S	850 m	15	40	3

*N*_m_, *N*_g_, *N*_c_ number of sampled individuals for morphometric, genetic and aromatic chemical analyses respectively.

### Genotyping

Three to four populations of *J. rossii* per habitat type were sampled between January and July 2012 in Réunion (sampling permit code from the Parc National de La Réunion: DIR/I/2012/002); that is, a total of ten populations and 410 plant individuals (see Table 1 and Fig. 1 for sampling sizes and distribution of populations respectively). For each plant, leaf material was desiccated in the field with silica gel, and DNA was later extracted using a DNeasy® Plant mini kit (Qiagen, Hilden, Germany). Individuals were genotyped for 13 polymorphic microsatellite loci–namely P2G7, P1A9, P2E3, P2H10, P2G6, P2G11, P2G2, P2E12, P2D1, P1B10, P2E2, P2G4 [26], plus a new locus P2G8 (F: 5′-CAGCCGAGAGAGTGTGTGAG-3′, R: 5′-GACCATGCTGTCGGAATTTT-3′) designed during this study. PCR multiplexes with fluorescently labelled primers were performed as in [26]. PCR fragments were resolved by capillary electrophoresis on an automated sequencer ABI Prism 3100 Genetic Analyzer (Applied Biosystems). Allele sizes were determined using Genemapper analysis software (Applied Biosystems).

### Genetic diversity

All pairs of loci were tested for linkage disequilibrium using a probability test in Genepop 4.0 [27]. Critical significance levels for multiple testing were corrected applying a sequential Bonferroni correction. FreeNA [28] was used to estimate null allele frequencies, for each locus in each population, according to the expectation maximization (EM) algorithm of Dempster et al. [29]. The mean observed number of alleles per locus (*A*_L_) and the number of private alleles (*A*_P_) per population were computed using GenAlEx 6.5 [30], [31]. Allelic richness (*A*_R_, El Mousadik and Petit [32]), as implemented in the software FSTAT 2.9.3 [33], was used to make direct comparisons of the mean number of alleles among populations regardless of sample size. Expected heterozygosity (*H*_E_) over all loci, observed heterozygosity over all loci (*H*_O_), multilocus *F*_IS_ estimated through the fixation index of Weir and Cockerham [34] were calculated using Genepop 4.0 [27]. The exact tests of Guo and Thompson [35] based on Markov chain iteration were used to test for departures from Hardy-Weinberg Equilibrium (HWE). To test for differences in amount of genetic variability (*A*_R_ and *H*_E_) between habitats, a test of comparison among groups of populations using FSTAT 2.9.3 [33] was performed with 9999 permutations.

### Genetic differentiation

Analysis of molecular variance (AMOVA; [36]) was performed using GenAlEx 6.5 to determine the relative partitioning of total genetic variation among habitat types, among populations of a same habitat, and within populations. The multilocus fixation index (*F*_ST_) was computed among pairs of populations as in Weir and Cockerham [39], and statistical significance was tested by 1000 random permutations of genotypes among populations using Genepop 4.0. To evaluate whether stepwise mutations contribute to the genetic differentiation between populations or between habitat types, a test developed in Hardy et al. [37] was performed from the microsatellite data. Based on a randomization of allele sizes among allelic states (1000 permutations), this test computes an *R*_ST_ (an analogue of *F*_ST_ based on allele size rather than allele identity; [38]) among populations, and can be interpreted as testing whether *F*_ST_ = *R*_ST_. To compare our results on genetic differentiation in *J. rossii* with other allozyme-based population studies in orchids [20], Hedrick’s *G’*_ST_ [39]–a standardized estimator of population genetic differentiation– was calculated using GenAlEx 6.5 [30], [31]. This estimator is particularly well suited for highly polymorphic microsatellites for which *F*_ST_ is, by definition, inferior to the average within-population homozygosity, even when no alleles are in common between subpopulations [39]. To overcome this problem, *G’*_ST_ is defined in order to have the same range, 0–1, for all levels of genetic variation. To determine if genetic drift occurs in the population differentiation of *J. rossii*, the effective population size (*N*e) of each population and migration rates between populations were estimated. *N*e of each population was estimated using a single-sample method (as opposed to temporal methods that require at least two data sets from the same population). LDNE software [40] was used to calculate *N*e from linkage disequilibrium in the data set. For this analysis, random mating was assumed, and all alleles with frequencies lower than 0.05 were excluded from the analyses. To estimate recent migration rates among populations and to identify which factor (geographic or environmental distances) affect migration among populations, a Bayesian method based on microsatellite multilocus genotypes was applied, as implemented in BIMr [41]. This software uses Bayesian assignment to infer the proportion of recent immigrants in a population from their genotypes and calculates corresponding asymmetrical migration rates between pairs of populations. Then, migration rates were related to factors in a generalized linear model. Five runs of MCMC were carried out and the obtained migration rates correspond to the average of the five runs. For each run, 5,040,000 iterations were used. The first 20,000 iterations consisted of short pilot runs used to tune up the proposal distributions to obtain reliable acceptance rates. The next 20,000 iterations were discarded as burn-in and the remaining observations were sampled every 50 iterations, giving a sample size of 100,000 for each analysis.

### Genetic structure

Assignment of multi-locus genotypes to different clusters was examined using two methods. Following a Bayesian clustering method, we ran InStruct [42] for K = 1 to K = 12 genetic clusters in mode 4 in order to infer the genetic structure and inbreeding coefficients. Whereas Structure [43] minimizes deviations from HWE within an inferred population, InStruct considers inbreeding or selfing rate in the model. For each value of K, InStruct was run with ten independent chains, each chain being run along one million iterations with a burn-in of half a million and a thinning interval of ten steps. To determine the optimal K, mean log-likelihood of the data [43] and ΔK [44] were plotted for each K. An alternative method, implemented in the adegenet package 1.3–4 [45] for R 2.15.1 [46], using K-means clustering of principal components for K = 1 to K = 39 and Bayesian Information Criterions was performed to assess the best number of genetic cluster. The two dissimilar approaches were used in this study, because different clustering approaches may lead to different conclusions [47], [48]. In order to test whether the genetic differentiation is structured by habitats, population structure was also explored by performing Discriminant Analysis of Principal Components (DAPC; [49]) with habitats as grouping factor. DAPC analysis is a recent multivariate approach that does not make any assumption about HWE or linkage equilibrium. DAPC transforms genotypes using PCA as a prior step to a discriminant analysis. The latter is performed to a number of principal components retained by the user (60 representing 84% of total genetic variation in this study) in order to maximize the among-population variation and minimize the variation within predefined groups [49]; that is, habitat types in the present case. DAPC was applied using the adegenet package 1.3–4 [45] for R 2.15.1 [46].

### Differentiation in floral morphology

Flowers were sampled from eight out of the ten populations under study during the flowering season in 2012 (see Table 1 for sampling details). In each population, one to four mature flowers were harvested from 11 to 25 randomly sampled individuals for a total of 133 individuals and 326 flowers, and stored in 70% ethanol (sampling permit code from the Parc National de La Réunion: DIR/I/2012/002). For each flower, ten traits were measured using a digital calliper (to 0.01 mm): spur length, column height, lip length and width, lateral petal length and width, adaxial sepal length and width, lateral sepal length and width. Differences in each floral trait among habitat types (with population as a nested factor) were investigated using an ANOVA followed by a Bonferroni corrected pairwise t-test. Differences in overall floral traits among habitat types (with population as a nested factor) were investigated using a MANOVA followed by a Canonical Discriminant Analysis (CDA) to describe morphological differentiation among habitat types in a multivariate space. This method maximizes the separation between pre-defined groups (i.e. habitat types in this case), and identifies the most explaining variables that separate the group centroids. Combination of traits contributing to each canonical variate was inferred from the magnitude and sign of structure coefficients associated with each floral trait. These statistical analyses were computed using R 2.15.1 [46].

### Differentiation in floral scent

Volatiles emitted by flowers were sampled from six out of the ten studied populations during the flowering season in 2012, from a total of 16 individuals (see Table 1 for sampling details). In the field, we selected intact plants and flowers and sampled their volatiles using a dynamic headspace method. A stem bearing three freshly opened flowers was enclosed in a polyacetate bag (19×19×24 cm) soon after dusk (18.00–19.00 h), after which the air was pumped out from the bag for 60 min at 200 mL/min through a quartz tube (15 mm long; 2 mm diameter) containing a 1∶1 mixture of 3 mg Tenax-TA (mesh 60–80, Supelco) and Carbotrap (mesh 20–40, Supelco) using a portable membrane pump (Spectrex PAS-500). A negative control was obtained repeating the same procedure with a stem bearing no flower. Scent samples were subsequently analysed by direct mass spectrometry (MS) coupled to gas chromatography (GC) analyses as described in [50]. The GC-MS data were processed using MS Worksation 7 Software. Compounds were identified thanks to the library NIST 02 mass spectral through a comparison of the retention times with published data [51]. Differences in relative emission rate of the 10 major compounds among habitat types (with population as a nested factor) were investigated using a permutational MANOVA (based on non-normal distributions).

### Geographical and environmental distances between populations

Geographical distances between populations were calculated from their GPS coordinates. For environmental distances between populations, seven variables that characterize each population were used (altitude, monthly mean minimum temperature, monthly mean maximum temperature, annual rainfall, annual number of rainy days, maximum daily rainfall and Emberger’s pluviothermic quotient [52]). Climatic variables were provided by Météo-France and were collected by the nearest weather stations from populations. Nine stations were available, one for each population and the same for Bébour and Bébour-Takamaka. These data correspond to climate means over several years, from six to twenty-nine according stations. A principal components analysis was performed on these seven variables and brings together populations according to their respective habitats (Fig. S1). This result indicates that the principal components can be used as a proxy to quantify habitat heterogeneity. Principally temperatures and altitude separated populations from submountain and mountain forests while rainfalls separated populations from windward and leeward forests (Fig. S1). Then, Mahalanobis distances between populations were calculated on the first two principal components.

### Effect of environmental and geographical factors on genetic and phenotypic divergence among populations

Differentiation in floral morphology and scent between populations was estimated using pairwise Mahalanobis D^2^, calculated in the principal component space. Genetic differentiation between populations was estimated using pairwise *F*_ST_ values. We first examined independently the role of geographic (IBD) and environmental distances (IBE) in genetic differentiation using Mantel tests. Significance of coefficients and R^2^ were estimated after 9999 random permutations completed in GenAlEx 6.5 [30], [31]. To analyse the relative role of environment (IBE) and geography (IBD) in phenotypic and genetic differentiation, we then used a new method introduced by Wang [11] using multiple regression analysis on matrices of genetic, morphological, chemical, geographic, and environmental distances. This approach allows quantifying how a dependent variable (genetic, morphology, chemistry) responds to changes in several explanatory variables (geography and environment). We applied the R function “MMRR” (Multiple Matrix Regression with Randomization, [11]) independently on genetic, morphological and chemical distance to obtain the regression coefficients and significance values for all parameters after 9999 random permutations. To exactly calculate the relative importance of the two explanatory distance matrices on the dependent matrix, these should not be related [11]. This is verified using a Mantel test after 9999 permutations.

## Results

### Genetic diversity

The analysis of 13 microsatellite loci in 10 populations and 410 individuals of *J. rossii* revealed high levels of genetic variability within populations, with mean numbers of alleles per locus (*A*_R_) ranging from 5.57 to 9.27 and expected heterozygosities (*H*_E_) from 0.680 to 0.793 (Table 2). Level of genetic diversity in terms of *A*_R_ and *H*_E_ was higher in MLR than in SWR (Table 2; *P* = 0.002 and *P* = 0.0005 respectively) but not significantly different for the other pairs of habitats. All populations showed private alleles (*A*_P_), from 1 to 8 according to the population (Table 2). However, only 17 out of 46 private alleles had a frequency >0.02, whereas only 4 had a frequency >0.05. *F*_IS_ estimates range from 0.164 to 0.523, and exact tests showed a significant deviation from HWE due to a heterozygote deficiency in all populations (Table 2). No pair of loci in disequilibrium was observed, suggesting that all loci are independent. The average frequency of null alleles resulted 0.08±0.10.

**Table 2:** Estimates of genetic diversity at 13 microsatellite loci in 12 populations of Jumellea rossii and means per habitat type.

Population/*Habitat*	*N*	*A*_L_	*A*_R_	*A*_P_	*H*_E_	*H*_O_	HWE	*F*_IS_
PF	23	8.46±3.55	8.34±3.48	5	0.758±0.241	0.405±0.159	***	0.469
BB	50	9.38±3.10	8.02±2.57	4	0.748±0.148	0.394±0.148	***	0.477
BBT	48	9.08±3.45	7.80±2.75	1	0.759±0.195	0.636±0.195	***	0.164
IG	52	9.23±3.11	7.89±2.29	4	0.756±0.151	0.469±0.151	***	0.383
CL	40	10.38±3.55	8.83±2.96	3	0.791±0.173	0.476±0.136	***	0.400
SP	50	9.23±3.17	8.35±2.76	3	0.772±0.136	0.397±0.184	***	0.490
MK	34	11.00±3.76	9.27±3.05	8	0.793±0.184	0.596±0.173	***	0.250
MR	21	5.62±1.71	5.57±1.70	1	0.680±0.156	0.328±0.156	***	0.523
DG	52	9.62±3.66	7.88±2.93	5	0.724±0.149	0.490±0.149	***	0.325
RBV	40	9.15±3.16	7.85±2.57	6	0.715±0.152	0.443±0.152	***	0.382
*MWR*	*173*	*9.04±0.40*	*8.01±0.24*	*3.5*	*0.755±0.005*	*0.476±0.112*	***	*0.373*
*MLR*	*124*	*10.20±0.90*	*8.82±0.46*	*4.7*	*0.785±0.012*	*0.490±0.100*	***	*0.380*
*SWR*	*113*	*8.13±2.19*	*7.10±1.33*	*4.0*	*0.706±0.023*	*0.420±0.083*	***	*0.410*
Total/mean	410	9.12±1.42	7.98±0.97	4.1	0.750±0.035	0.463±0.094	***	0.386

*A*_L_, mean number of alleles per locus ± s.d.; *A*_R_, mean allelic richness per locus ± s.d.; *A*_P_, private allelic richness; *H*_E_, expected heterozygosity over all loci ± s.d.; *H*_O_, observed heterozygosity over all loci ± s.d.; HWE, result of test for departures from Hardy–Weinberg Equilibrium, ***P<0.001; *F*_IS_, fixation index of Weir and Cockerham [34]; MWR, mean per population of mountain windward rainforest; MLR, mean per population of mountain leeward rainforest; SWR, mean per population of submountain windward rainforest.

### Genetic differentiation

According to the AMOVA, 93.5% of the total genetic variation was found within populations, only 4.4% among populations of a same habitat type, and 2.1% among habitat types. The average pairwise *F*_ST_ across population was 0.040±0.011 ranging from 0.023 to 0.063. All values were highly significant and indicated a low genetic differentiation among populations. When allele sizes were taken into account, the global genetic differentiation *R*_ST_ = 0.073 was not significantly different than that based on allele identities, global *F*_ST_ = 0.056 (*P*>0.05). The global Hedrick’s standardized *G’*_ST_ was 0.231 among all populations. Genetic differentiation was slightly but significantly higher (Wilcoxon rank test, *W* = 342.5, *P* = 0.0002) among populations of different habitat types (*G’*_ST_ = 0.233±0.048 ranging between 0.154 and 0.315) than among populations of a same habitat (*G’*_ST_ = 0.150±0.029 ranging between 0.112 and 0.201).

The mean effective population size (*N*_e_) across populations was 32 individuals with a minimum of 6 in Mourouvain (95% CI: 3–8) and a maximum of 91 in Basse-Vallée (95% CI: 57–199). In addition to Basse-Vallée, only Cilaos had a *N*_e_ of more than 40 individuals (*N*_e_ = 75; 95% CI: 55–112). The results from five different runs with the software BIMr were concordant and estimated that the mean immigration rate between each pair of subpopulations was 3.91×10^−3^ with a minimum of 9.88×10^−11^ and a maximum of 1.05×10^−1^. The numbers of recent migrants per generation (*N*_m_) between each pair of subpopulations were obtained by multiplying the effective size of the population of origin by migration rates (Table 3). All *N*_m_ values were less than one with two exceptions: 8.06 migrants per generation from Basse-Vallée to Dugain and 1.81 from Saint-Paul to Cilaos. Moreover, the best model explaining the migration rates among populations was the one including only environmental distances as an explanatory variable (Table 4). The regression coefficient for the effect of environment was negative; migration rates are thus reduced among populations from different environments (Table 4).

**Table 3:** Estimated number of recent migrants (Nm) per generation between populations of Jumellea rossii.

Into\From	PF	BB	BBT	IG	SP	MK	CL	MR	DG	RBV
**PF**	36.90	1.0×10^−8^	1.5×10^−8^	1.0×10^−8^	7.5×10^−9^	3.8×10^−9^	3.9×10^−8^	1.6×10^−9^	5.7×10^−9^	3.5×10^−8^
**BB**	1.3×10^−8^	16.20	2.6×10^−8^	4.4×10^−9^	3.6×10^−9^	2.4×10^−9^	2.0×10^−8^	1.0×10^−9^	3.1×10^−9^	2.4×10^−8^
**BBT**	7.6×10^−9^	6.4×10^−9^	30.40	2.6×10^−9^	1.9×10^−9^	1.1×10^−9^	1.1×10^−8^	5.6×10^−10^	1.6×10^−9^	1.1×10^−8^
**IG**	1.3×10^−8^	3.4×10^−9^	6.3×10^−9^	17.00	6.2×10^−9^	3.2×10^−9^	3.9×10^−8^	8.0×10^−10^	2.5×10^−9^	1.7×10^−8^
**SP**	1.2×10^−8^	4.1×10^−9^	9.5×10^−9^	8.4×10^−9^	17.10	3.5×10^−9^	4.1×10^−8^	1.8×10^−9^	3.5×10^−9^	2.1×10^−8^
**MK**	9.9×10^−9^	2.8×10^−9^	5.5×10^−9^	7.9×10^−9^	5.5×10^−9^	11.30	2.7×10^−8^	6.3×10^−10^	3.2×10^−9^	2.2×10^−8^
**CL**	0.67	0.19	0.59	0.21	1.81	0.44	59.12	0.02	0.04	0.30
**MR**	7.9×10^−9^	4.7×10^−9^	6.7×10^−9^	3,0×10^−9^	2.2×10^−9^	1.4×10^−8^	1.2×10^−8^	5.60	6.4×10^−9^	3.3×10^−8^
**DG**	0.16	0.39	0.04	0.04	0.02	0.03	0.09	0.07	13.03	8.06
**RBV**	2.1×10^−8^	6.6×10^−9^	1.7×10^−8^	6.8×10^−9^	5.3×10^−9^	4.7×10^−9^	2.9×10^−8^	2.3×10^−9^	9.5×10^−9^	91.30

Nm were obtained by multiplying the effective size of the population of origin by recent migration rates per generation. The direction of migration is given from populations in columns (origin) to those in lines (destination).

**Table 4:** Posterior model probabilities for models explaining migration rates among populations of Jumellea rossii.

		Regression coefficients estimated [95% HPDI]		
Factors included	Model probability	α1	α2	α3
**None**	0.201			
**Geography**	0.112	−0.30 [−2.32; 2.29]		
**Environment**	**0.465**		−**0.77** [−2.96; 2.90]	
**Geography and** **environment**	0.162	−0.26 [−2.54; 2.14]	−0.82 [−2.84; 2.95]	
**With interaction**	0.060	−0.32 [−2.38; 1.97]	−0.54 [−2.56; 2.76]	0.23 [−2.05; 1.71]

Values represent means of the 10 runs.

### Genetic structure

**Figure 2:**
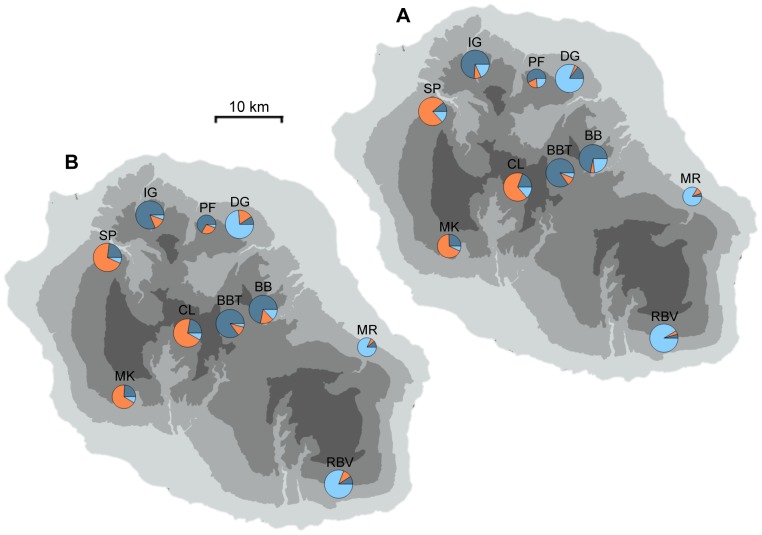
Spatial genetic structure of *Jumellea rossii* populations obtained by two different methods. Genetic structure is inferred (A) by a model-based clustering method implemented in InStruct and (B) by discriminant analysis of principal components with habitat type as grouping factor. At each location, pie charts indicate the mean proportion of individual memberships in each cluster for K = 3 (A) or each habitat type (B) and their size is proportional to the number of individuals sampled.

Clustering of microsatellite genotypes using InStruct algorithm showed that the distribution of ΔK with increasing K presented two modal values; the higher is located at K = 2 and the second, much lower, at K = 3 (Fig. S2). K determination using K-means clustering of principal components and the Bayesian Information Criterion revealed K = 3 as the best number of cluster to use (Fig. S2). Moreover, K = 3 seemed to be the best model to describe our data and appeared to be better than K = 2 to reflect biological processes because it also corresponded to the number of different habitats. The population genetic structures at K = 3 using Bayesian clustering (InStruct) and the results of DAPC (adegenet) are shown in Figure 2. InStruct clearly distinguished three clusters according to habitat types (Fig. 2A), all populations in SWR forming the first, all populations in MLR the second, and all populations in MWR the third. Despite an apparent genetic structure, there was admixture between all clusters. When the three genetics clusters are defined a priori by habitat type (DAPC, Fig. 2B), assignment analyses reveal the same pattern as described above.

### Differentiation in phenotypic traits

**Figure 3:**
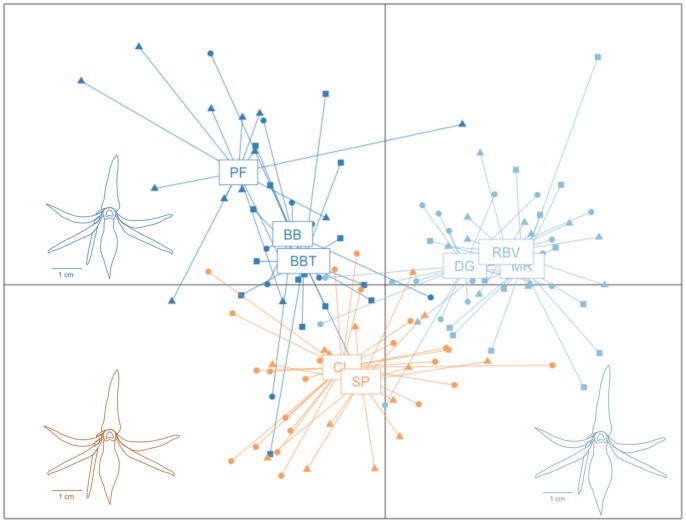
Canonical discriminant analysis of 10 morphological floral traits with habitat type as grouping factor. Flowers of one population of each habitat (PF, CL and RBV) are drawn. The colours correspond to the type of habitat, dark blue for the mountain windward rainforest, light blue for the submountain windward rainforest and orange for the mountain leedward rainforest.

The MANOVA was highly significant for habitats and populations within habitats (*P*<0.0001 in both cases), indicating that significant morphological differences exist among habitats and among populations within habitat. This variation could be effectively visualized using the two first canonical variables of the CDA for habitat comparisons (Fig. 3). At the habitat level, CV1 and CV2 accounted all of the variation, describing 69.6% and 30.4% of the variation, respectively. CV1 separated SWR from the two other habitats principally based on lip width and lateral petal width with respectively positive and negative coefficients. CV2 primarily described adaxial sepal width differences with a negative coefficient, distinguishing MWR and MLR. Means and standard deviations from measurements of ten floral characters in *J. rossii* are shown in Table 5. All traits varied significantly among habitat types and all except adaxial sepal width varied significantly among populations of a same habitat type. However, only spur length, lip width and adaxial sepal width were more variable between habitats than between populations in the same habitat. Spur length and lip width were the most variable traits between habitat types: they were significantly smaller in SWR than in MWR or MLR but no significant difference was observed between MWR and MLR.

**Table 5:** Comparison of the ten measured floral traits of Jumellea rossii among habitats [mean±s.d.].

Floral trait	Habitat			Nested ANOVA	
	MWR*(N = 40)*	MLR*(N = 42)*	SWR*(N = 51)*	Habitat	Population within habitat
Spur Length	20.49±1.79^a^	20.64±2.31^a^	18.28±1.97^b^	***23.5%	*6.8%
Column Height	2.26±0.71^a^	2.01±0.28^b^	2.03±0.18^b^	**6.9%	***15.3%
Lip Width	6.62±0.57^a^	6.59±0.57^a^	7.26±0.61^b^	***23.2%	**9.8%
Lip Length	21.40±1.62^a^	22.29±2.17^ab^	23.00±2.09^b^	***10.3%	***15.7%
Lateral Petal Width	3.39±0.44^a^	3.13±0.28^b^	3.22±0.29^b^	***10.2%	***42.3%
Lateral Petal Length	20.63±1.67^a^	21.77±2.52^b^	21.66±2.00^ab^	***13.1%	***18.2%
Adaxial Sepal Width	4.42±0.39^a^	4.78±0.40^b^	4.60±0.33^c^	***14.2%	NS 6.5%
Adaxial Sepal Length	19.87±1.70^a^	21.57±2.37^b^	21.80±2.16^b^	***14.6%	***16.4%
Lateral Sepal Width	3.55±0.42^a^	3.70±0.24^b^	3.76±0.28^b^	***8.2%	***36.7%
Lateral Sepal Length	21.01±1.77^a^	22.80±2.41^b^	22.69±2.17^b^	***5.4%	*** 16.3%

MWR, Mountain windward rainforest; MLR, Mountain leeward rainforest; SWR, Submountain windward rainforest. Means followed by the same letter at the same row are not significantly different (P<0.05) according to the pairwise t-test with Bonferroni correction, *P<0.05, **P<0.01, ***P<0.001.

In the analysis of floral volatiles, we identified ten major compounds across all 16 individuals examined (Table S1): that is, nine aromatic compounds and one monoterpene. The most common compounds were (in decreasing order) benzaldehyde, benzyl acetate, benzyl alcohol and eugenol, all found in any individuals. Other trace compounds were found in some individuals (Table S1). The permutational MANOVA was not significant for habitats and populations within habitats (*P = *0.91 and *P = *0.70 respectively), indicating that there was no significant difference in the floral volatiles among habitats and among populations within habitat.

### Effect of environmental and geographical factors on eenetic and phenotypic divergence among populations

**Figure 4:**
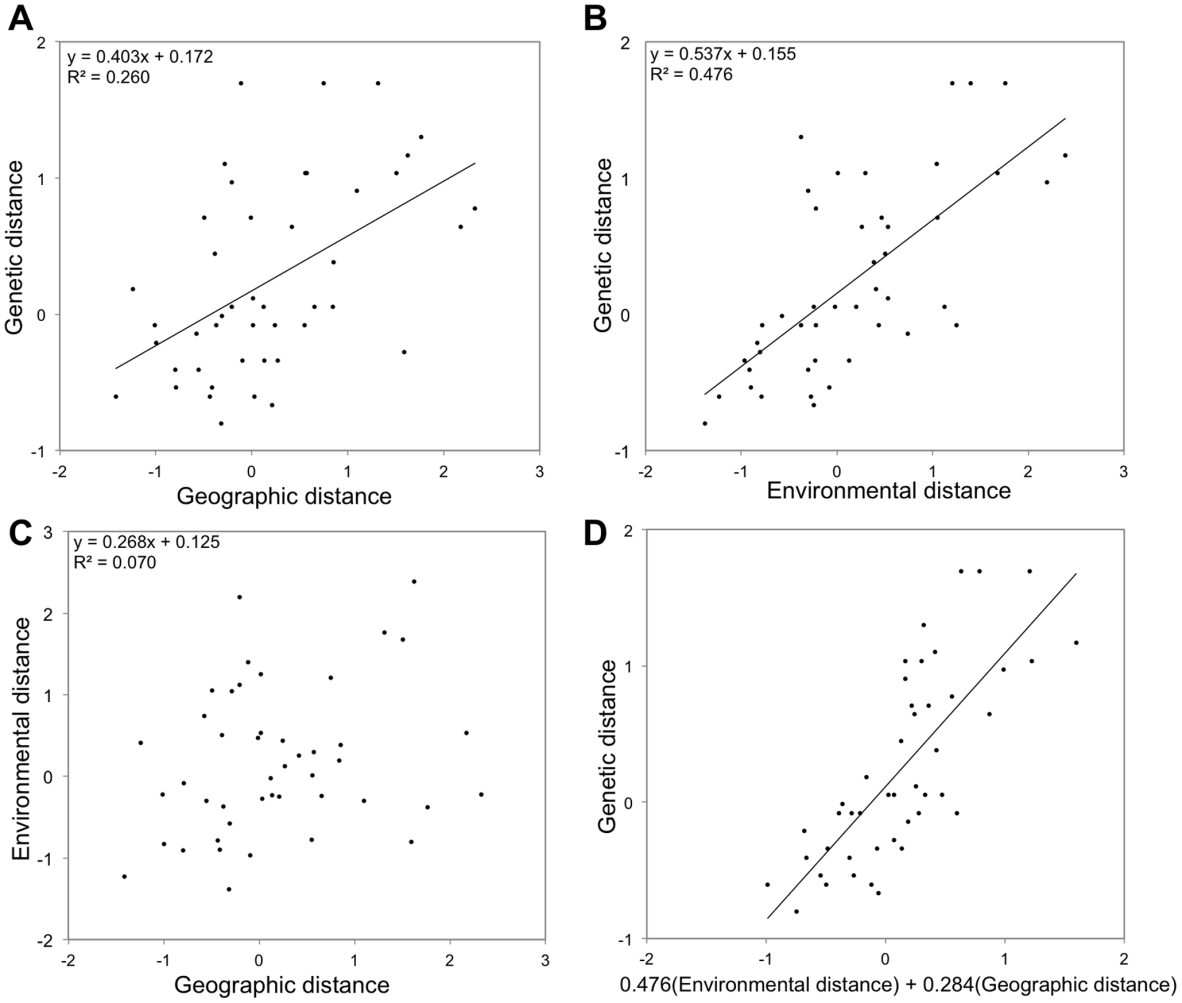
Multiple matrix regression with randomization (MMRR) analysis performed on genetic distances. Scatterplots show patterns of isolation-by-distance (A), isolation-by-ecology (B) and the absence of eco-spatial autocorrelation (C) according to [4]. When correlations are significant (Mantel test, P<0.05), regression lines are drawn. Plot (D) is based on the results of the multiple matrix regression analysis for the effects of both geographical and environmental distances on genetic distances.

Mantel tests between geographic distances and genetic distances and between environmental distances and genetic distances revealed that both IBD and IBE played a significant and independent role in genetic differentiation of populations. Indeed, environmental and geographic distances were both strongly associated with genetic distances but not significantly correlated with each other (Fig. 4A–C). However, the multiple matrix regression analysis, used to explore the relative role of environment (IBE) and geography (IBD) in genetic differentiation, pointed out that the regression coefficient for environmental distances (β_E_ = 0.476, *P* = 0.004) was about two times greater than the regression coefficient for geographic distances (β_G_ = 0.284, *P* = 0.043) suggesting that IBE explained the majority of genetic distance (Fig. 4D). Concerning the phenotypic differentiation, only the geographic distances contributed to the morphological differentiation (β_G_ = 0.385, *P* = 0.038) compared to environmental distances, which did not have a significant role (β_E_ = 0.357, *P = *0.076). Neither geographical distances nor environmental distances significantly explained the chemical distances between populations.

## Discussion

### Isolation-by-habitat as a primary factor causing population differentiation in *J. rossii*

We found a significant genetic differentiation between populations of the epiphytic orchid species *Jumellea rossii* at relatively small geographic scale i.e. less than the total surface area of Réunion (2,500 km^2^). Genetic clustering analyses showed that the general pattern of population differentiation in *J. rossii* was structured geographically in relation with the variation in forest type. Moreover, multiple matrix regression analyses also indicated that the three main forest types where *J. rossii* was found, and which were characterized by seven environmental variables in the analyses, primarily caused population differentiation in this orchid species compared to geography. In other words, gene flow seems to occur preferentially among populations of a similar habitat type. Conversely, gene flow seems to occur less frequently between populations of different habitat types, even if these are geographically close. This was well illustrated by the two populations growing on eastern slopes of the island at Plaine des Fougères (1220 m) and Dugain (770 m): although only 5 km separate them, we observed a significant genetic differentiation between these two populations (*G*’_ST_ = 0.186) that mainly differed in respect to the forest type. The general pattern of isolation-by-habitat was also supported by a Bayesian analysis revealing that the best model explaining the migration rates among populations was the one based on the environmental variables only. Thus, migrations among populations whose environmental characteristics are dissimilar appear to be less frequent.

Pattern of genetic differentiation obtained with microsatellites markers can be viewed as the result of stochastic evolutionary processes [53], and neutral genetic differentiation can thus suggest that random mechanisms (i.e. drift in combination with spatially restricted gene flow) underlie divergence between populations. Moreover, small effective populations sizes, less than one migrant per generation between populations and high inbreeding coefficients in *J. rossii* are the conditions under which genetic drift can occur [1], [54]. In flowering plants, gene flow and migration between populations are caused by pollen and/or seed dispersal. Orchids have dust-like seeds that are wind-borne and, therefore, would have the potential for long-distance dispersal. Nevertheless, recent studies demonstrated that orchid seeds establish better close to their mother plant [55]–[57], even for some epiphytic taxa [16]. Concerning pollen dispersal in *J. rossii*, one likely explanation for the observed pattern of isolation-by-habitat would be variation in the flowering phenology. Differences in flowering phenology between populations growing under different climatic conditions, e.g. in more or less elevated rainforests, should restrict gene flow through asynchronous pollination and ultimately cause genetic isolation. For instance, the two geographically close and genetically differentiated populations growing at Plaine des Fougères and Dugain blossomed in late December-early January and late February-early March respectively. Nevertheless, variation in flowering phenology alone cannot explain the overall pattern of genetic differentiation. Indeed, populations growing at the same altitude along the environmental gradient from east to west, i.e. in mountain windward rainforests or in mountain leeward rainforest respectively, flower synchronously. For example, the populations growing at Cilaos (1400 m) and at Bébour-Takamaka (1370 m) both blossomed in late December-early January. Moreover, as they are 8 km away from one another, they could have potentially exchanged genes through pollen flow. Despite the fact that these flowering populations coexist spatially and temporally, we observed a significant genetic differentiation (*G*’_ST_ = 0.219) between them. In this particular case, as in several other cases, we believe that gene flow may be limited by IBE. Under IBE, local adaptation of populations to their habitat drives their ecological niche differentiation and thus limits the likelihood of gene flow between them [4]. In plants, ecological isolation is mainly based on the selection against migrants [58], [59] i.e. pollinating insects and/or seed dispersal. Divergent selection between distinct environments is probably the best understood driver for population differentiation and speciation [60] and has been proposed as a major contributing factor to explain the diversification of species in environmentally heterogeneous ecosystems such as rainforests [61]. In the study area i.e. Réunion, IBE was also suggested as the main factor causing local population differentiation in a passerine bird [14].

### Is phenotypic differentiation consistent with genetic differentiation?

We previously showed that both IBD and IBE contributed to the pattern of genetic differentiation among populations of *J. rossii* but that the latter factor was more likely to explain it. Under IBE, the proximal cause of population differentiation is ecologically-based divergent selection [4], [60] which can arise, in flowering plants, from pollinator preferences for specific phenotypic floral traits [60], [62]. Differentiation of floral morphology among populations of *J. rossii* was concordant with genetic differentiation and was also structured by habitat. For example, lip width tends to decrease along the gradient from east to west, and spur length tends to increase with altitude. Even if such consistent patterns of morphological variation can theoretically arise through random processes [1], this scenario becomes unlikely when many populations are considered and natural selection or phenotypic plasticity are more realistic explanations. Studies that have addressed phenotypic selection in natural orchid populations have shown that interactions with pollinators can lead to selection on floral traits that influence pollination efficiency, notably the spur length [63]–[65]. However, knowledge on the distribution, relative abundance and behaviour of pollinators of *J. rossii* in each habitat type is too scarce to support the hypothesis of pollinator-mediated divergence. Moreover, in this species, floral volatiles did not differ significantly between populations or between habitats. Floral odour is highly relevant for pollinator attraction and is consequently expected to be under strong selection, especially in night pollinated flowers [66]. A comparative study of the variation in floral scent between two closely related orchids demonstrated that the overall variation was significantly lower in the rewarding species than in the deceptive one, suggesting a stabilizing selection imposed by floral constancy of the pollinators in the rewarding species [67]; this might also be the case in *J. rossii*. Moreover, spatio-temporal variation in pollinators of a generalist plant might likely result in inconsistent selective regimes that will greatly reduce the possibilities of local adaptation to particular pollinators [68]. *Jumellea rossii* has a generalist pollination system because three species of effective pollinators have been identified so far but their relative abundance in each habitat type, their influence on the orchid reproductive success and the dispersal distance of pollen remains to be investigated. Our results suggest that specific plant-pollinator mutualism does not play a major role in the population differentiation of *J. rossii* but that other factors are involved. In this case, phenotypic plasticity could be a likely explanation for the morphological differentiation of populations. Plasticity of floral traits has indeed been documented in response to variation in environmental factors such as water, light, temperature and nutrient availability [69]–[71]. Furthermore, phenotypic plasticity in floral morphology can influence the strength and direction of pollinator-mediated natural selection. For example, if floral traits respond plastically to abiotic environment, pollinator-mediated selection on these traits may differ between different habitats [72].

### Are populations of *J. rossii* undergoing speciation?

We found that populations of *J. rossii* were genetically structured according to the forest types and interpreted it as the result of habitat filtering in combination with variation in flowering phenology. One question here remains: are populations of *J. rossii* undergoing speciation on Reunion Island? From a broader perspective, the relationship between population divergence and speciation is not always obvious. Indeed, some species can maintain high population differentiation without necessarily splitting into several lineages [73]. Conversely, speciation can sometimes emerge in the presence of gene flow [74], [75], in particular when the diverging populations become highly adapted to their respective habitats [76], [77].

Although populations of *J. rossii* are genetically differentiated, there is no clear evidence that this orchid species is undergoing speciation. However, genetic differentiation between populations has long been perceived as an early step during the speciation process [78]–[81] and reduction in gene flow i.e. reproductive isolation is critical prior to speciation [82]. Moreover, intra-island cladogenesis appears to be recurrent on elevated remote oceanic islands, possibly because colonization rate is low, ecological space is initially unsaturated and ecological heterogeneity is high [13], [83] particularly when the maximum topographic complexity is reached [13]. In Réunion, there are strong evidences that altitudinal gradient and habitats diversity promote partition among species. First, habitat type coupled with altitude influence orchid species composition and the distribution of orchid breeding systems [24]. Second, ecological speciation along altitudinal and environmental gradients is strongly suggested by phylogenetic studies that have identified intra-island radiations in various plant taxa, such as for instance the genera *Psiadia* [84] and *Dombeya* [85]. From a single event of colonization from Madagascar to Reunion, possibly through some Mauritian members that went extinct, theses genera have repeatedly undergone speciation along the turnover of forest types found on Réunion [84], [85]. In the orchid genus under study i.e. *Jumellea*, we observe the same tendency in the phylogeny [25], [86] notably in the clade of *J. rossii* that diverged from the lowland forest species *J. fragrans* through adapting to the cloudy mountain and submountain rainforests. Further research on the genetic differentiation among populations of *J. fragrans* and *J. rossii* will help understanding the mechanisms driving speciation in this orchid clade and perhaps answering the question on whether populations of *J. rossii* are undergoing speciation.

## Supporting Information

Figure S1 **Principal components analysis of environmental variation between populations of** ***Jumellea rossii*** **.** Based on altitude (Alt), monthly mean minimum (Tmin) and maximum (Tmax) temperatures, annual rainfall height (ARH), annual number of rainy days (ARD), maximum daily rainfall (MDR) and Emberger’s pluviothermic quotient (EPQ). The colours correspond to the type of habitat, dark blue for the mountain windward rainforest, light blue for the submountain windward rainforest and orange for the mountain leedward rainforest.
(TIF)

Figure S2 **Detection of the number of genetic clusters K.** (A) Using K-means algorithm and the Bayesian Information Criterion (BIC) for each K with adegenet [49]. (B) Using the log-likelihood (triangles) and ΔK statistic according to Evanno et al. [44] (squares) averaged over ten runs for each K with InStruct [42].
(TIF)

Table S1 **Mean relative abundances of major volatile organic compounds found in floral scent of** ***Jumellea rossii*** **populations.**
(PDF)
