# Supplementary figures and images for: Evidence for Isolation-by-Habitat among Populations of an Epiphytic Orchid Species on a Small Oceanic Island
Source: PLoS One. 2014 Feb 3;9(2):e87469. doi: 10.1371/journal.pone.0087469 (PMC3911949; doi:10.1371/journal.pone.0087469)

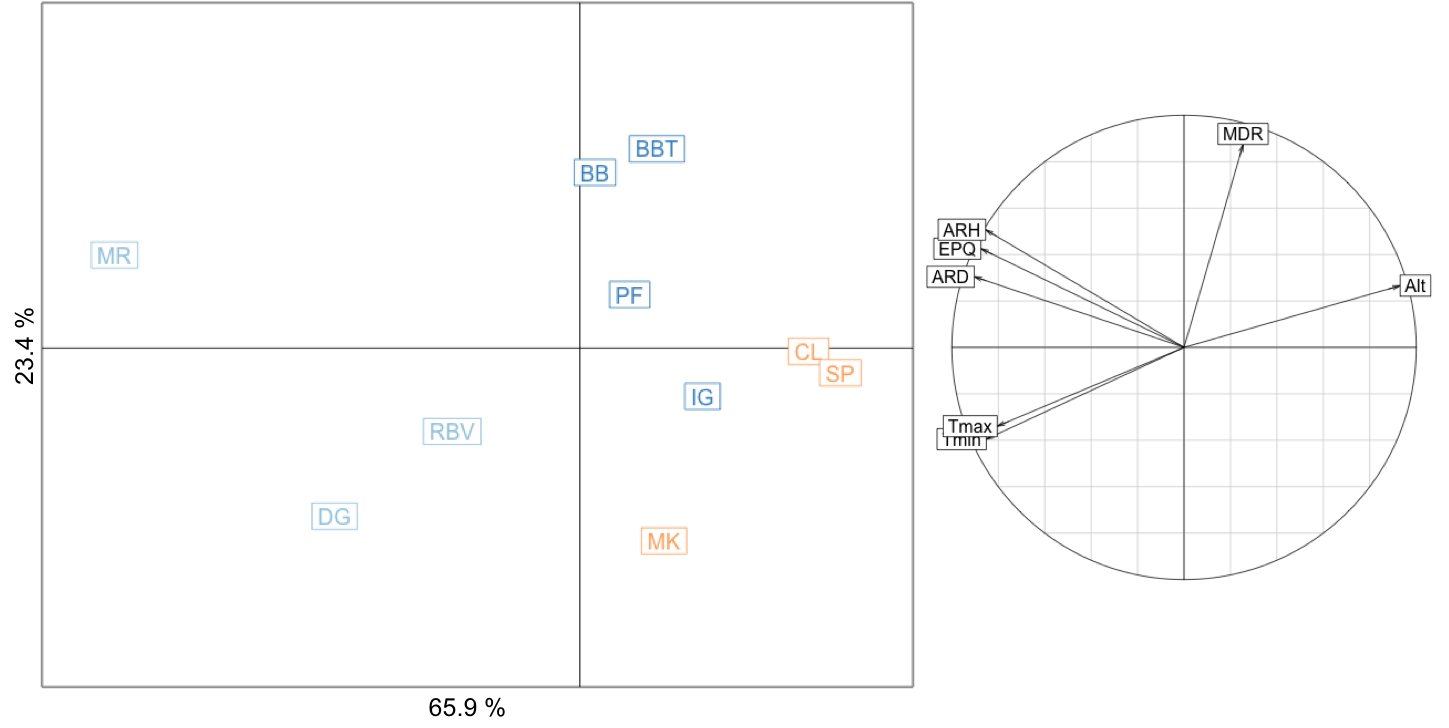

Supplement: Figure S1 — Principal components analysis of environmental variation between populations of Jumellea rossii . Based on altitude (Alt), monthly mean minimum (Tmin) and maximum (Tmax) temperatures, annual rainfall height (ARH), annual number of rainy days (ARD), maximum daily rainfall (MDR) and Emberger’s pluviothermic quotient (EPQ). The colours correspond to the type of habitat, dark blue for the mountain windward rainforest, light blue for the submountain windward rainforest and orange for the mountain leedward rainforest. (TIF) [file pone.0087469.s001.tif]

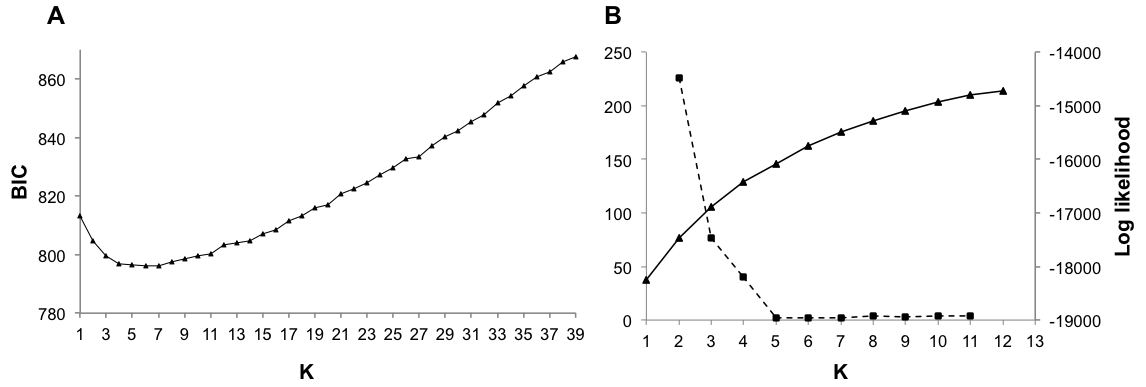

Supplement: Figure S2 — Detection of the number of genetic clusters K. (A) Using K-means algorithm and the Bayesian Information Criterion (BIC) for each K with adegenet [49]. (B) Using the log-likelihood (triangles) and ΔK statistic according to Evanno et al. [44] (squares) averaged over ten runs for each K with InStruct [42]. (TIF) [file pone.0087469.s002.tif]
