# Supplementary material for: Evidence for Isolation-by-Habitat among Populations of an Epiphytic Orchid Species on a Small Oceanic Island
Source: PLoS One. 2014 Feb 3;9(2):e87469. doi: 10.1371/journal.pone.0087469 (PMC3911949; doi:10.1371/journal.pone.0087469)
Supplement: Table S1 — Mean relative abundances of major volatile organic compounds found in floral scent of Jumellea rossii populations. (PDF) [file pone.0087469.s003.pdf]

Table S1. *Mean relative amounts of the volatile compounds identified in the floral scent of different population of* Jumellea rossii *emitted at dusk.*

Compounds	T_R[a]	BBT[b]	CL[b]	SP[b]	DG[b]	MR[b]	RBV[b]
**Monoterpenes**							
Limonene	8.621	2.18	0.00	0.00	1.46	0.00	0.02
**Aromatics**							
Anisole	10.381	5.48	0.00	0.12	0.58	0.00	0.00
Benzaldehyde	12.684	47.42	60.62	42.57	44.71	53.36	61.46
Benzyl alcohol	16.713	15.83	16.31	25.04	20.10	15.26	10.30
Benzyl acetate	15.058	21.97	12.83	25.01	15.36	16.31	16.42
Benzyl benzoate	23.608	0.00	0.00	0.00	0.36	0.03	0.28
Chavicol	21.059	0.22	0.15	0.15	0.59	1.02	0.38
Dichlorobenzene	11.633	0.25	0.00	0.00	3.01	0.09	0.00
Eugenol	19.579	6.65	10.08	7.10	13.41	13.66	11.42
Vanillin	23.885	0.00	0.02	0.00	0.43	0.28	0.00

(a) T_R : Retention times on apolar column SPB-5.
(b) Relative percentages calculated from the peak areas of the chromatogram
